# Sterol Composition in the Lichens *Lobaria pulmonaria* and *Lobaria retigera*: Does Photobiont Matter?

**DOI:** 10.3390/ijms262211041

**Published:** 2025-11-14

**Authors:** Julia N. Valitova, Venera R. Khabibrakhmanova, Vasiliy M. Babayev, Ajsylu F. Khajrullina, Oleg P. Gurjanov, Natalia I. Gazizova, Richard P. Beckett, Farida V. Minibayeva

**Affiliations:** 1 Kazan Institute of Biochemistry and Biophysics, FRC Kazan Scientific Center of RAS, P.O. Box 261, Kazan 420111, Russia; yulavalitova@mail.ru (J.N.V.); venerakhabirakhmanova@gmail.com (V.R.K.); a16280110@gmail.com (A.F.K.); gurjanov58@gmail.com (O.P.G.); natgazizova@mail.ru (N.I.G.); rpbeckett@gmail.com (R.P.B.); 2 Department of Food Biotechnology, Kazan National Research Technological University, Kazan 420015, Russia; 3 Arbuzov Institute of Organic and Physical Chemistry, FRC Kazan Scientific Center of RAS, Kazan 420088, Russia; babaev@iopc.ru

**Keywords:** lichen, *Lobaria pulmonaria*, *Lobaria retigera*, photobiont, mycobiont, sterols, saponification

## Abstract

The lipid composition of the mycobint and photobiont symbiotic partners of lichenized ascomycetes varies greatly. The aim of this study was to compare the profile of the major sterols in two closely related lichens from the genus *Lobaria* with different photobionts. The three-component lichen *Lobaria pulmonaria* has two photobionts. While the main photobiont is the chlorophycean alga *Symbiochloris reticulata*, this lichen contains small amounts of the cyanobacterium *Nostoc*. By contrast, the cyanobacterium *Nostoc* is the main photobiont in *Lobaria retigera*. Relatively loosely bound sterols were extracted using a chloroform–methanol mixture, and subsequently, more tightly bound sterols by alkaline saponification. The initial chloroform–methanol extraction step indicated that ergosterol is the principal sterol in both species, with phytosterols constituting a minor fraction. However, the addition of an alkaline saponification step to the standard protocol of sterol extraction greatly increases the release of tightly bound phytosterols, such as campesterol, stigmasterol, and β-sitosterol from *L. pulmonaria,* but not from *L. retigera*. Therefore, the mycobionts and *Nostoc* mainly possess sterols extractable by the standard mixture of chloroform/methanol, while the chlorophycean algal photobiont contains tightly bound sterols. This observation could be important when studying the roles of sterols in the stress tolerance of lichens.

## 1. Introduction

Lichens are a symbiotic association of a fungal mycobiont, usually an *Ascomycete*, and a green algal (chlorophycean) or cyanobacterial photobiont. The sterol composition of lichens is rich and diverse and varies significantly depending on the type of lichen and its symbionts [1]. Typically, 95% of the lichen biomass is mycobiont, and only 5% is photobiont. Both myco- and photobionts synthesize sterols, and they may influence the transport of solutes and metabolites between the symbionts by modulating membrane permeability [2]. While ergosterol is the main sterol of the mycobiont, other sterols, such as lichesterol, episterol, fungesterol, and other ergosterol derivatives, may also be present in minor quantities [3]. By contrast, green algal photobiont of lichens predominantly contains phytosterols, although minor quantities of other sterols may also be present. For example, Whittemore showed that the green alga *Trebouxia* isolated from the lichen *Xanthoria parietina* contains 24-ethylcholesta-5,22E-dien-3β-ol (stigmasterol) and 24-methylcholesta 5en-3β-ol (campesterol) [4]. While earlier studies suggested that cyanobacteria are unable to synthesize sterols, their presence was first reported in 1968 [5,6]. Unsaturated sterols such as 24-ethyl sterols were isolated from the filamentous cyanobacterium *Phormidium luridum* [6]. Later, sterols, including cholesterol, 24-ethylcholest-5-en-3β-ol, Δ7 and Δ5,7-sterols, were reported in cyanobacteria [7]. It has been shown that 70–80% of the sterols produced by the cyanobacterium *Nostoc carneum* are cholesterol, while the rest comprise isofucosterol, 22-dehydrocholesterol, and stigmastanol [8]. Recently, Fagundes et al. [9] found the presence of β-sitosterol, stigmasterol, and cholesterol in cyanobacterium *P. autumnale.*

Application of the standard lipid extraction protocol to lichen thalli involving chloroform and methanol would suggest that the great majority of lichen sterols are produced by the mycobiont. However, earlier works showed that two pools of sterols exist. The first pool comprises the “solvent extractable” sterols, i.e., those extracted by organic solvents such as chloroform and methanol, and second, the “tightly bound” sterols, which can be further extracted using alkaline saponification [10,11]. It has been shown that alkaline saponification of the lichen *X. parietina* after exhaustive solvent extraction releases a tightly bound sterol fraction with a significantly different composition. Comparison of the solvent-extractable and tightly bound sterols of *X. parietina* strongly suggests that organic solvents extract sterols from the mycobiont, whereas saponification extracts more tightly bound sterols from the photobiont [10].

The aim of this study was to compare the profile of the major sterols in two closely related lichens from the genus *Lobaria*, *L. pulmonaria* and *L. retigera*. While the mycobionts of these species are very closely related, they contain different photobionts. The tripartite (three-component) lichen *L. pulmonaria* has two photobionts; the main photobiont is the green alga *Symbiochloris reticulata*, but this lichen contains small amounts of the cyanobacterium *Nostoc* in internal structures called cephalodia. By contrast, in the lichen *L. retigera*, the main photobiont is the cyanobacterium *Nostoc*. The task was to analyze whether adding alkaline saponification to the standard extraction procedure can facilitate the release of not only chloroform–methanol extractable sterols but also tightly bound sterols from lichens with different photobionts. This approach is necessary to elucidate more comprehensively the sterol profiles from the myco- and photobionts.

## 2. Results

The yield of lipids and the sterol profiles of the three extracts of the lichens *L. pulmonaria* and *L. retigera* were analyzed. The three lipid extracts were as follows:

(I) extracts of lichen tissue prepared by the Bligh–Dyer protocol;

(II) extracts prepared by the Bligh–Dyer protocol subjected to saponification;

(III) extracts of residual lichen tissue subjected to saponification.

Gravimetrical analysis showed that yields of lipophilic substances extracted from *L. pulmonaria* and *L. retigera* by the chloroform–methanol (I) were similar, being 23 ± 1 and 20 ± 2 mg g^−1^ dry mass (DM), respectively. After saponification of the chloroform–methanol lichen extracts (II), yields were 4.9 ± 1 mg g^−1^ DM for *L. pulmonaria* and 3.0 ± 0.8 mg g^−1^ DM for *L. retigera*. Alkaline saponification of residues (III) increased the yield of unsaponifiable substances five times in *L. pulmonaria* (14.6 ± 3.5 mg g^−1^ DM), but only slightly in *L. retigera* (3.7 ± 1.8 mg g^−1^ DM).

**Figure 1:**
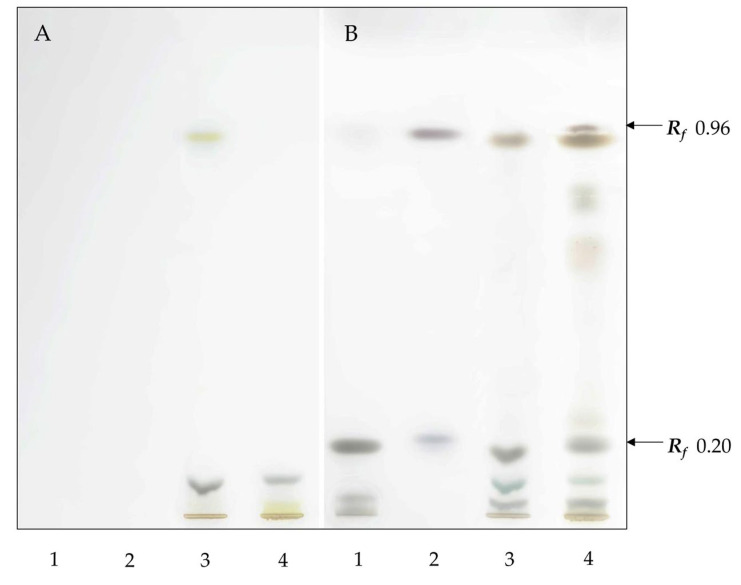
Visualization of lichen sterols extracted by the Bligh–Dyer protocol and separated by HPTLC, before (**A**) and after (**B**) treatment with FeCl_3_; 1—ergosterol (8 μg), 2—cholesteryl stearate (8 μg), 3—extract of *Lobaria retigera* (100 μg); 4—extract of *Lobaria pulmonaria* (100 μg).

On a high-performance thin-layer chromatography (HPTLC) plate treated with FeCl_3_, bands were visible in the lane containing lichen extracts corresponding to ergosterol (*R_f_* 0.2) and also with a dark purple coloration with a similar Rf to the standard for the sterol ester cholesteryl stearate (*R_f_* 0.96) (Figure 1). A purple-colored band was clearly visible in the extract from *L. pulmonaria*, while in the lane with extract from *L. retigera,* only a very faint band that could only be seen by visual inspection was present. Brown bands with slightly lower *R_f_* in the lanes contain lichen extracts which do not correspond to the sterol ester and presumably correspond to other compounds, e.g., waxes.

The relative contents of the major sterols typical for the mycobiont (ergosterol) and the photobiont (β-sitosterol, campesterol, stigmasterol) were assessed using the ratio between individual peak areas and to the total sum of the peak areas of all identified sterols on the gas chromatography-mass spectrometry (GC-MS) chromatogram.

**Figure 2:**
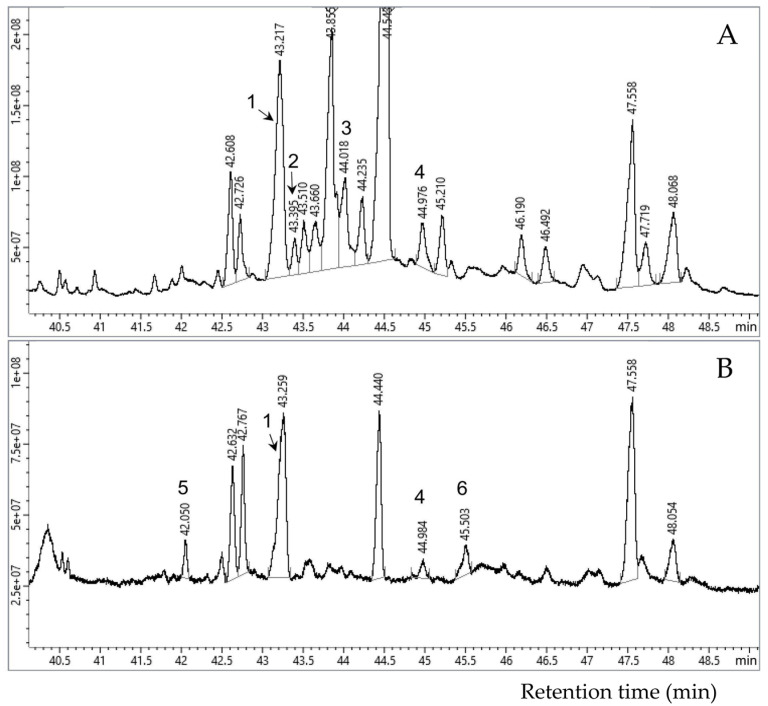
Representative GC-MS chromatograms of silylated free sterols from *Lobaria pulmonaria* (**A**) and *Lobaria retigera* (**B**). Peaks: 1—ergosterol; 2—campesterol; 3—stigmasterol; 4—β-sitosterol; 5—cholesterol; 6—lanosterol.

Analysis of the sterol profile of *L. pulmonaria* showed that lipid extract (I) comprised mainly the fungal sterol ergosterol, with much lower proportions of the plant sterols *β*- sitosterol, campesterol, stigmasterol (Figure 2). Saponification of this extract (II) increased the proportion of stigmasterol, while the proportion of ergosterol decreased. The proportions of campesterol and β-sitosterol remained at the same levels (Table 1). The alkaline saponified-extract from the residual lichen tissue (III) comprised a much greater proportion of plant sterols, i.e., stigmasterol and campesterol, and much less of the fungal ergosterol (Table 1).

Analysis of the sterol profile of *L. retigera* showed that extract (I) contained mainly ergosterol and minor quantities of cholesterol and β-sitosterol. After saponification, in extract (II) the proportion of ergosterol slightly decreased, the proportions of cholesterol and β-sitosterol increased, and the triterpenoid lanosterol was detected (Table 2). After alkaline saponification of residual lichen tissue, extract (III) contained similar proportions of ergosterol and β-sitosterol as extract (II), but cholesterol was absent in this fraction (Table 2).

## 3. Discussion

The aim of this study was to compare the profile of the major sterols in two closely related lichens from the genus *Lobaria*, *L. pulmonaria* and *L. retigera* with different photobionts. The standard sterol extraction protocol was modified by the addition of an alkaline saponification step. Results of using the classical extraction protocol show that the fungal sterol ergosterol is the major sterol in both *L. pulmonaria*, a lichen with a mainly chlorophycean photobiont, and *L. retigera*, a lichen with a cyanobacterial photobiont. However, the addition of a saponification step increases the yield of phytosterols in *L. pulmonaria* but not *L. retigera*. Our findings suggest that using only the classical chloroform–methanol extraction has limitations in the study of lichen sterols, but the addition of saponification yields a more comprehensive profile for chlorophycean species.

Currently, the generally accepted method for isolating sterols from lichen thalli, and also other tissues, is the Bligh–Dyer method that uses organic solvents such as chloroform and methanol. However, it is becoming clear that to thoroughly determine all sterols present in lichens, a sequential extraction technique is required. It is necessary to subject residues remaining after the initial extraction to an alkaline saponification. This is especially important for lichens because their thalli comprise several different organisms, i.e., the fungal mycobiont and one or more photobionts that are cyanobacterial or chlorophycean. Differences in the membrane systems of the constituent symbiotic partners may lead to differences in the extractability of their sterols. The earlier work of Lenton [10] showed that while organic solvents readily extract mycobiont sterols from *X. parietina*, alkaline saponification is required to extract a second, more tightly bound pool of sterols from the photobiont. However, *X. parietina* only contains one photobiont, a green alga belonging to the genus *Trebouxia*. Here, for the first time, we use the approach of Lenton to analyze a chlorophycean lichen and a closely related cyanobacterial lichen. One species is *L. pulmonaria*, in which the main photobiont is the chlorophycean photobiont *S. reticulata*, but that also contains small amounts of the cyanobacterium *Nostoc* in cephalodia. The second species is *L. retigera* that, in contrast, has *Nostoc* as its only photobiont.

In the lichen *L. pulmonaria*, application of the standard Bligh–Dyer protocol shows that the main sterol is fungal ergosterol, with only small amounts of the plant sterols campesterol, stigmasterol and β-sitosterol present (Table 1). Apparently, the chloroform–methanol readily extracts mycobiont sterol. Interestingly, saponification of the initial extracts from *L. pulmonaria* increases the proportion of stigmasterol from 3% to 24% (Table 1). This may indicate that stigmasterol is present in algal cells mainly in the form of esters and conjugates, because saponification primarily breaks ester bonds [12]. To our knowledge, the presence of stigmasterol in the cyanobacterium *Nostoc* has not been shown so far. It is possible that stigmasterol esters are important in the regulation of various aspects of membrane function [13].

Alkaline saponification of the residues of *L. pulmonaria* remaining after the first extraction further extracts phytosterols such as stigmasterol and campesterol and a small amount of ergosterol (extract III, Table 1). Our data are consistent with those of Lenton [10], who showed that the algal sterols of *X. parietina* occur in a tightly bound fraction. It seems likely that the photobiont sterols are less readily extracted due to their presence in symbiotic algal cells of tough cell walls, which are often thicker than those of their free-living counterparts [2]. Cell-wall thickening may serve as a defense mechanism against fungal penetration and help regulate the symbiotic interface, ensuring a stable and balanced symbiosis.

As in *L. pulmonaria*, the major sterol in *L. retigera* is fungal ergosterol (Table 2). It is known that ergosterol has high reactivity due to the presence of a system of conjugated double bonds in the ring (ref). In fungi, ergosterol plays extremely important roles in respiration and oxidative phosphorylation in mitochondria, and therefore, it has been suggested that this sterol can be considered as a metabolic marker [14].

In contrast to *L. pulmonaria*, the lipid extract of *L. retigera* obtained using the Bligh–Dyer protocol involving chloroform and methanol contains significant amounts of cholesterol and β-sitosterol, characteristic of *Nostoc*, in addition to the usual fungal sterols (Table 2). In free-living *N. carneum,* it has been shown that the predominant sterols are cholesterol, β-sitosterol, and stigmastanol [8]. Interestingly, after saponification of the initial lipid extracts, the triterpenoid lanosterol was detected (Table 2). The absence of this compound in the original extract indicates that lanosterol naturally occurs in lichens as a derivative. Some fungi contain a variety of triterpenoid derivatives, including lanosterol. Triterpenoids are known to display a broad spectrum of biological activity [15,16]. Saponification also increases the proportion of cholesterol and β-sitosterol compared to the original extract, indicating that a fraction of these sterols occurs naturally as esters. Alkaline saponification of the lichen tissues remaining after the initial extraction with organic solvents has no significant effect on the yield or the sterol composition of the alkaline extract (Table 2), suggesting that extraction with organic solvents alone is sufficient for cyanolichens.

Comparison of the phytosterols present in both lichens shows that although both lichens contain β-sitosterol, these species differ in other phytosterols. For example, unlike *L. pulmonaria*, *L. retigera* contains significant amounts of cholesterol. Cholesterol is known to have a significant effect on permeability of artificial membranes, while campesterol, β-sitosterol, and stigmasterol have a lesser effect [13]. Therefore, it can be suggested that the presence of cholesterol in the sterol pool of the lichen *L. retigera* makes cell membranes more stable. Furthermore, stigmasterol and campesterol are present in *L. pulmonaria* but not *L. retigera*. In plants, stigmasterol is considered a “stress” sterol, as its content in tissues increases in response to various types of stress [13]. The presence of stigmasterol and its esters in the sterol pool of *L. pulmonaria* facilitates a range of membrane-modulating adaptation strategies for this lichen in stress conditions.

Results of experiments using saponification suggest that in the initial lichen extracts, stigmasterol in *L. pulmonaria* and cholesterol and β-sitosterol in *L. retigera* are mostly present as esters with higher fatty acids. Free sterols are rigid, flat molecules that are tightly packed with phospholipid fatty acid chains, reducing membrane fluidity and permeability [17]. In contrast, sterol esters, with their bulky fatty acid chains, cannot be packed as tightly, and therefore, their incorporation into the membrane leads to “packing defects” and increases local membrane fluidity. The balance between free sterols and sterol esters helps to regulate the formation, stability, and signaling functions of photobiont membranes [18,19].

## 4. Materials and Methods

### 4.1. Lichen Material

The lichen *L. pulmonaria* (L.) Hoff. was collected from trunks of aspen trees (*Populus tremuloides* Michx.) in woodlands on the outskirts of Syktyvkar, Komi Republic, Russia (N61.936, E50.743). Material of *L. retigera* was collected from branches of *Leucosidea sericea* Eckl. and Zeyh., a small tree that grows in Afromontane forest in Monks Cowel in the province of KwaZulu-Natal, South Africa (N29.415, E29.915). *L. pulmonaria* is a three-component lichen comprising two photobionts, the cyanobacteria *Nostoc punctiforme* (Kütz.) Har. and the green alga *Symbiochloris reticulata*. Thalli were air-dried at room temperature and 60% relative air humidity (RH) between sheets of filter paper and stored at −20 °C until use.

### 4.2. Lipid Extractions

Three types of extracts from the lichens *L. retigera* and *L. pulmonaria* were obtained:

Extract (I). In the first stage, lipophilic compounds were extracted from 1 g sample of dry lichen using a mixture of chloroform and methanol solvents (1:2) according to the Bligh–Dyer method [20]. The chloroform fraction was dried on a rotary evaporator (IKA RV 8, Staufen im Breisgau, Germany) and the yield of lipophilic substances was determined by the gravimetric method [21]. Extracts were stored at −20 °C until use for analysis.

Extract (II). For alkaline hydrolysis, a 1 M KOH solution in 80% methanol (based on 4 mL of saponifying agent per 20 mg of extractive substances) was added to the dry residue of the lichen lipophilic extract, mixed, and heated in a water bath (IKA HB, Staufen im Breisgau, Germany) at 80 °C for 30 min. Then, 4 mL of diethyl ether and 6 mL of distilled water were added to the reaction mixture, mixed, and left to separate into layers (30 min, 4 °C). This procedure was repeated twice. The upper ether layer was separated and combined with the first-stage ether extract. The total ether extract was washed with distilled water in 5 mL portions, discarding the lower aqueous layer, and this procedure was repeated three times. Then, anhydrous sodium sulfate (0.5 g) was added to the total ether extract, mixed, kept for 10 min and then filtered. The ether from the extract was evaporated on a rotary evaporator at a temperature not exceeding 30 °C. As in our preliminary experiments using HPTLC with a specific solvent system, we did not detect glycosylated forms of sterols, so it was unnecessary to perform acidic hydrolysis of the initial lichen extracts [21].

Extract (III). To extract tightly bound sterols, lichen residues remaining after the first stage of extraction were subjected to alkaline saponification. Residues were transferred to a round-bottomed flask, 20 mL of a methanol-distilled water–KOH mixture (80:10:10) added, and the mixture was heated under reflux for 2 h at 95 °C. The hydrolysate was separated by centrifugation after preliminary cooling. Distilled water and diethyl ether in the ratio 1:2 were added, the mixture was thoroughly mixed, and then left to separate at 4 °C under reflux for 2 h. The saponification mixture was cooled, diluted with H_2_O (1 volume) and partitioned with diethyl ether (3 volumes), dried over Na_2_SO_4_ and solvent removed to yield the non-saponifiable lipids. The total diethyl ether extract was washed with distilled water three times in 5 mL portions. The purified extract was filtered through anhydrous sodium sulfate (1 g) to remove residual water. The ether from the extract was evaporated on a rotary evaporator at a temperature not exceeding 30 °C.

The yield of substances isolated all stages was determined gravimetrically.

### 4.3. HPTLC Analysis of Lipid Extracts (I)

HPTLC was carried out in an automated CAMAG system (Muttenz, Switzerland). Merck KGaA HPTLC Silica gel 60 glass plates (1.05641.0001, Darmstadt, Germany) were used for chromatography. Lichen extracts prepared by the Bligh–Dyer protocol were dissolved in chloroform–methanol (1:1) (12.5 mg/mL). To identify the sterols and their esters, we used a standard—ergosterol, cholesteryl stearate (Sigma-Aldrich, St. Louis, MO, USA, purity no less than 98%). Extracts and standards were applied to the plate using a Linomat 5 automatic applicator in the form of a 7 mm-wide band. Elution was carried out in an ADC 2 automatic chamber, the front line of the mobile phase was 80 mm. Hexane:diethyl ether:acetic acid (80:20:1) was used as the mobile phase. Next, TLC plates were sprayed with solution of ferric chloride using a spray bottle (Lenkhrom, Saint-Petersburg, Russia), dried, and heated for 2–3 min at 100 °C.

### 4.4. GC-MS Analysis of Sterols

Dry residues of the extracts (I, II, III) were dissolved in pyridine with the addition of hydrocarbon C23 (Tricosan) (1 mg mL^−1^) as a quality control, converted into trimethylsilyl derivatives using BSTFA, and the mixture was heated for 15 min at 100 °C. The resulting silylated derivatives were analyzed using GC-MS system “Chromatec-Crystal 5000” (ZAO SKB “Chromatec”, Yoshkar-Ola, Russia).

Parameters of ionization method: electron ionization (70 eV); ion source temperature 200 °C; mass range: 50–650 amu.; capillary column BP-5MS 30 m long, 0.25 mm internal diameter, 0.25 μm phase film thickness.

Gas chromatography separation conditions: injected sample volume—0.5 μL; initial temperature of the column thermostat—70 °C, thermostatting at the initial column temperature for 2 min, temperature increase at a rate of 6 °C/min to 300 °C, thermostatting at the final column temperature for 10 min; sample injection unit (injector) temperature—280 °C; sample injection mode with flow split 5:1, carrier gas-helium, flow rate—1 mL/min.

Identification of sterols was carried out using the AMDIS program, NIST 17 mass spectral libraries and corresponding sterol standards ergosterol, cholesterol, stigmasterol (Sigma-Aldrich, St. Louis, MO, USA, purity no less than 98%) [11] (Table 3).

The article presents the average data of three independent series of experiments. The tables and graphs show arithmetic averages and their standard deviations. The data were analyzed using ANOVA followed by using the Duncan criterion at a significance level of *p* < 0.05.

## 5. Conclusions

The results of this study demonstrate that for lichens relying solely on the classic Bligh–Dyer method may lead to an inaccurate estimation of sterol composition, because the method does not always fully extract photobiont sterols. This is of particular importance when the main lichen photobiont is chlorophycean. It seems likely that the membrane sterols of some photobionts are difficult to extract, firstly because the photobiont layer is embedded in fungal tissue, and secondly because the photobiont cells typically have thick cells walls with a mucilaginous sheath. Lichens with chlorophycean photobionts require an additional extraction step involving alkaline saponification of the residues remaining after the initial exaction. Chlorophycean photobionts contain mainly plant sterols, and according to the terminology of Lenton [10], can be classified as tightly bound sterols. In contrast, sterols of the mycobiont and the cyanobacterial photobiont *Nostoc* are almost completely extracted by chloroform and methanol, and therefore, can be classified as readily extractable sterols. Despite the fact that more than 90% of lichens contain chlorophycean algae rather than cyanobacteria as their main photobiont [22], most studies on the responses of sterols to stress in lichens have only measured what is readily extractable, i.e., mycobiont and cyanobacterial sterols. As the photobiont is particularly sensitive to unfavorable environmental factors, future studies on the role of sterols in stress tolerance in lichens should use protocols that include extraction of the tightly bound sterols from the algal component.

## Figures and Tables

**Table 1:** Sterol composition of lichen *Lobaria pulmonaria* (% *, *n* = 3, values given ± s.d.).

Compound (Peak Number on the Chromatogram)	Solvent-Extractable Sterols		Tightly Bound Sterols
	(I) Chloroform–Methanol Extract	(II) Chloroform–Methanol Extract Followed by Saponification	(III) Extract from Lichen Residue After Saponification
Ergosterol (1)	73.3 ± 12.0 ^a^	55.6 ± 7.2 ^b^	18.7 ± 9.5 ^c^
Campesterol (2)	9.1 ± 1.0 ^a^	9.3 ± 3.0 ^a^	31.8 ± 4.4 ^b^
Stigmasterol (3)	3.1 ± 0.7 ^a^	24.7 ± 4.2 ^b^	49.5 ± 5.6 ^c^
β-Sitosterol (4)	14.5 ± 1.3 ^a^	10.4 ± 0.8 ^b^	-

* Total identified sterols taken as 100%. The mean values from three experiments and their standard deviations are presented. Significance of differences between the means are indicated by different letters (ANOVA, followed by Duncan’s multiple range test, *p* < 0.05).

**Table 2:** Sterol and triterpenoid composition of lichen *Lobaria retigera* (%, *n* = 3, values given ± s.d.).

Compound (Peak Number on the Chromatogram)	Solvent-Extractable Sterols		Tightly Bound Sterols
	(I) Chloroform–Methanol Extract	(II) Chloroform–Methanol Extract Followed by Saponification	(III) Extract from Lichen Residue After Saponification
Cholesterol (5)	0.7 ± 0.3 ^a^	4.3 ± 2.9 ^b^	-
Ergosterol (1)	98.0 ± 3.1 ^a^	91.0 ± 4.8 ^b^	97.2 ± 2.4 ^a^
β-Sitosterol (4)	1.3 ± 0.2 ^a^	4.7 ± 1.7 ^b^	2.8 ± 0.9 ^ab^
Lanosterol * (6)	-	5.1 ± 3.3	-

* Proportion of this triterpenoid is calculated from the sum of identified sterols. The mean values from three experiments and their standard deviations are presented. Significance of differences between the means are indicated by different letters (ANOVA, followed by Duncan’s multiple range test, *p* < 0.05).

**Table 3:** GC-MS data for the trimethylsilyl sterol esters (standards).

Compound	RI	Qualitative Fragments (*m/z*) *
Cholesterol	3147	129 (100), 213 (8), 255 (10), 329 (45), 353 (20), 368 (26), 443 (8), 458 (28)
Ergosterol	3229	131 (28), 211 (18), 253 (12), 337 (25), 363 (60), 378 (20), 468 (35)
Campesterol	3250	129 (100), 213 (6), 255 (4), 343 (50), 367 (20), 382 (35), 472 (25)
Stigmasterol	3276	83 (100), 129 (75), 213 (4), 255 (25), 355 (6), 394 (20), 484 (25)
β-Sitosterol	3337	129 (100), 213 (4), 255 (4), 357 (45), 381 (18), 396 (30), 486 (25)

RI—relative retention index based on interpolation of retention times of C10-C38 Alkanes Standard. * Original spectral data.

## Data Availability

The original contributions presented in this study are included in the article. Further inquiries can be directed to the corresponding author.
